# An In-Depth Study of Vibration Sensors for Condition Monitoring

**DOI:** 10.3390/s24030740

**Published:** 2024-01-23

**Authors:** Ietezaz Ul Hassan, Krishna Panduru, Joseph Walsh

**Affiliations:** IMaR Research Centre, Munster Technological University, V92 CX88 Tralee, Ireland; letezaz.ul.hassan@research.ittralee.ie (I.U.H.); joseph.walsh@mtu.ie (J.W.)

**Keywords:** predictive maintenance, vibration based condition monitoring, vibration data collection, vibration data sources

## Abstract

Heavy machinery allows for the efficient, precise, and safe management of large-scale operations that are beyond the abilities of humans. Heavy machinery breakdowns or failures lead to unexpected downtime, increasing maintenance costs, project delays, and leading to a negative impact on personnel safety. Predictive maintenance is a maintenance strategy that predicts possible breakdowns of equipment using data analysis, pattern recognition, and machine learning. In this paper, vibration-based condition monitoring studies are reviewed with a focus on the devices and methods used for data collection. For measuring vibrations, different accelerometers and their technologies were investigated and evaluated within data collection contexts. The studies collected information from a wide range of sources in the heavy machinery. Throughout our review, we came across some studies using simulations or existing datasets. We concluded in this review that due to the complexity of the situation, we need to use more advanced accelerometers that can measure vibration.

## 1. Introduction

Monitoring is defined as a method of observing and tracking different factors, actions, or systems in order to acquire information about their performance [1], behavior, or status. Maintenance refers to the actions and processes that are carried out to keep equipment, machinery, systems, and assets in good working condition [2]. Monitoring and maintenance methods can be successfully combined [2] with one another to ensure reliable performance and longer service life for heavy machinery. This integrated approach aims to reduce downtime [3], maintenance costs [4], and catastrophic failures [5,6]. In a variety of industries, condition monitoring [7] is a technique used to evaluate the performance and health of machines [8], tools [9], or systems in real time or on a regular basis. The aim of condition monitoring is to identify any changes from the normal operation, detect possible issues or faults early, and stop unexpected failures [10] or breakdowns [4]. There are many types of condition monitoring [11], including predictive, preventive, and corrective maintenance [9].

Preventive maintenance, predictive maintenance, corrective maintenance, reliability maintenance and total productive maintenance [9] are some of the well-known maintenance strategies. Preventive maintenance, which is commonly used in industries including manufacturing, transportation [12], and energy, comprises regularly scheduled standard procedures that increase equipment life, reduce catastrophic failures, and assure reliability. Predictive maintenance uses real-time equipment monitoring [13] and data processing [4] via sensors to predict maintenance needs based on actual equipment status, lowering costs and downtime. When repair costs [14] are less than preventive maintenance costs, corrective maintenance [11] (also known as “run-to-failure”) is usually cost-effective for non-critical equipment. It is frequently used for cheap parts or backup systems with little impact on operations as a whole. With the objective of minimizing maintenance efforts, reliability-centered maintenance (RCM) [15], a systematic strategy that prioritizes maintenance based on the value of the equipment while taking costs [16], operational impact, and safety into account, is often used. Total Productive Maintenance (TPM) [17], depending on equipment performance to boost output, involves all employees in proactive equipment maintenance to prevent problems.

Predictive maintenance [11] is important in current maintenance methods, bringing numerous advantages to a wide range of organizations. Predictive maintenance [18] improves cost efficiency by shifting from set schedules to on-demand maintenance, lowering over-maintenance costs and lowering the risk of unplanned breakdowns [19]. Predictive maintenance reduces unplanned downtime by considering possible problems before they arise, maintaining continued production and meeting deadlines. Regular maintenance based on real equipment condition increases the useful life of equipment and assets, decreasing the need for frequent replacements and resulting in long-term cost savings. Predictive maintenance makes use of condition monitoring data, which provides significant insight into equipment performance [20], health, and behavior. This data-driven [21] strategy promotes informed decision making as well as ongoing development. Predictive maintenance minimizes the under or over-utilization of maintenance resources that are associated with traditional techniques, resulting in more effective resource allocation and cost savings.

Heavy machinery is huge, strong mechanical equipment designed for heavy operations that require a lot of power, strength, and dependability [22]. These machines are widely used in industries such as construction, mining, agriculture, forestry, manufacturing, and transportation. Heavy machinery works in handling heavy loads, working in difficult conditions, and completing tasks quickly. Excavators, bulldozers, cranes, loaders and mining equipment are some examples of heavy machinery. Heavy machinery [23] operates by mechanical, hydraulic, electrical, and electronic systems, which include engines for generating power and hydraulic systems for controlling actions like lifting and excavation. Breakdowns in heavy machinery can stop everything completely, resulting in significant amounts of downtime [24]. Predictive maintenance helps prevent the occurrence of these breakdowns by predicting possible problems before they become catastrophic failures. These machines are equipped with sensors [25,26,27,28,29] that continuously collect data on parameters like temperature [9,20,25,30], pressure [7,20] and vibration [21]. These data are then evaluated by software, which uses cutting-edge methods such as machine learning [31], previous data, and contextual information to construct predictive models that identify expected issues or maintenance requirements based on usage patterns and environmental factors.

Data collected by sensors which record oscillations or motions within a structure or system is known as vibration data. It collects information that is important in evaluating the condition [32] and functionality of machinery or systems, such as vibration patterns, amplitude, and frequency. Vibration sensors monitor the intensity and frequency of vibrations in industrial components with unusual patterns indicating possible mechanical problems such as imbalances [9,33], wear [9,25,27,34,35,36,37] or misalignment [9,38,39,40,41,42]. Accelerometers are used for collecting vibration data from heavy machinery. These sensors are carefully placed on machinery to record mechanical vibrations and transform them into electrical signals [4,25] for analysis as well as for condition monitoring and maintenance. Accelerometers can be divided into several types based on the features, including piezoelectric, PCB, wired or wireless, MEMS, or triaxial accelerometer; each one is designed to specific functionalities in field use. Uniaxial accelerometers [43] measure vibration in a single axis, whereas triaxial accelerometers [44] measure vibration in three axes. Wireless accelerometers work without physical connections and transmit data using wireless technology. Piezoelectric accelerometers produce electrical charges in response to mechanical vibration, showing how these sensors detect vibration in many different ways. With an in-depth understanding of the condition of the machinery provided by this data gathering and analysis, maintenance staff is better able to identify anomalies and problems and schedule timely maintenance [45] to avoid breakdowns and enhance equipment performance. Vibration-based condition monitoring has many advantages in evaluating the condition and efficiency of machines.

Several methods are used to process vibration data obtained from heavy machinery for doing condition monitoring [32]. Some methods use the Fast Fourier Transform (FFT) analysis to highlight frequencies associated with anomalies or problems in the machinery by converting vibration signals from the time domain to the frequency domain [46]. Wavelet analysis also allows examination at various time-frequency resolutions, which allows for specific issues or transient events [47]. The goal of envelope analysis is to identify high-frequency component fluctuation in order to detect gear corrosion or bearing defects [48]. Statistical techniques generate benchmark patterns and identify deviations that might correspond to possible problems [49]. Machine learning algorithms are trained to identify patterns in vibration data, enabling predictive maintenance by identifying prior indications of future issues [50]. In order to determine the frequencies of resonance or other anomalies, spectral analysis is also used for analyzing frequency content [51]. By creating virtual models of actual machinery, digital twins provide predictive maintenance, real-time monitoring, and simulation-based continuous improvement [52]. Because machine learning [53], deep learning [54], and reinforcement learning algorithms are so good for the detection of patterns and anomalies in vibration data, they may detect faults early and create maintenance schedules that are based on prior data analysis. The IoT integration and networked systems of Industry 4.0/5.0 allow autonomous decision making and real-time data collection from sensors, improving the precision and efficiency of heavy machinery condition monitoring [55]. With the aim of establishing predictive maintenance methods and reducing unplanned downtime, each approach provides different insights into the state of the machinery.

This article provides a dual-focused review in the field of condition monitoring of heavy machinery. First, it discusses the importance, challenges, requirements and effects of vibration-based condition monitoring for heavy machinery. Then, we review the variety of tools used to gather vibration data, which is a key element of a machine’s health monitoring. It also looks at the various devices that are producing that vibration data. This study serves as an initial study for a further evaluation of the methods used in processing data. By carefully investigating these important aspects, we aim to provide insights into efficient condition monitoring systems, ultimately improving machine efficiency and reliability.

## 2. Methodology

**Figure 1:**
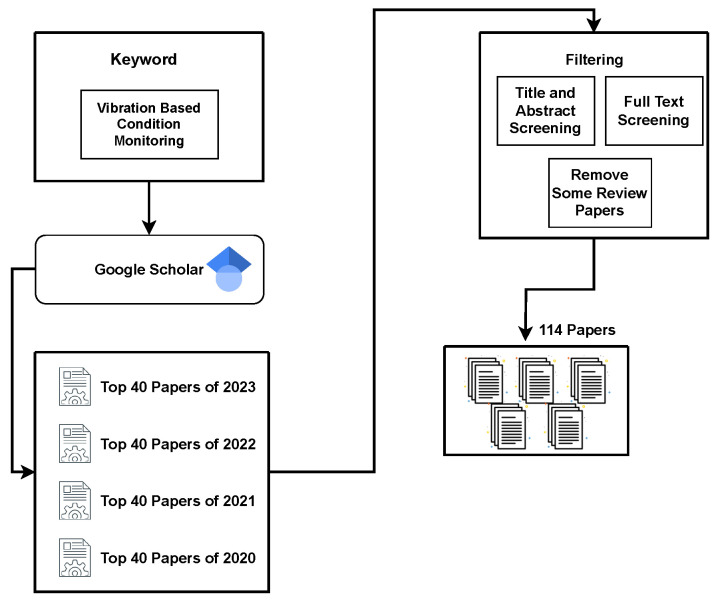
Flowchart of our work.

A systematic approach was used to conduct this literature review on vibration-based condition monitoring. Figure 1 shows the methodology used for this review. Initially, an in-depth search on Google Scholar was done using the keyword ‘vibration-based condition monitoring’ with a special focus on publications published in 2023, 2022, 2021, and 2020. We collected the top 40 articles from the first four pages of Google Scholar search results for each year. Precise filtering procedures were used to guarantee the relevance and quality of the papers that were chosen. The review method excludes some literature review papers in order to find the recent technologies used for collecting vibration data. Publications that did not meet the vibration-based condition monitoring standards in their title or abstract were also excluded. In addition, those papers that required a fee for access were identified and excluded. As a result, 114 papers were reviewed with a focus on exploring the device used for data collection, including conference, journal and transaction papers. This careful selection method made an effort to guarantee that the most relevant and reputable sources were included in our literature review analysis.

**Figure 2:**
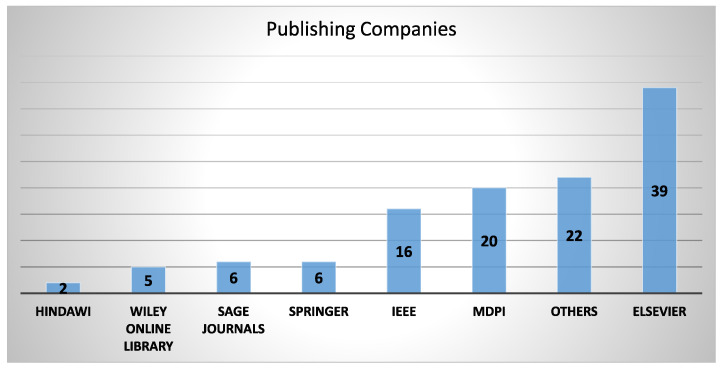
Distribution of papers by publishing company.

The results of this literature review included a wide range of published sources, each of which contributed to the full analysis of ‘vibration-based condition monitoring’. Figure 2 shows a breakdown of the distribution of articles among various publishing companies. Some of the most significant sources were IEEE (16 papers), Elsevier (39 papers), MDPI (20 papers), and Wiley Online Library (five publications). Additionally, Springer and Sage Journals each contributed six papers, whereas Hindawi contributed only two. There were 22 papers in the ’Other Sources’ category, including their contributions from: The Chinese Journal of Electronics, Journal of Engineering, Transportation Safety and Environment, ACS Publications, Design and Technology (JDMD), Sound and Vibration, Arxiv, Clean Energy, Biblio Urgent, ProQuest, Eawe and Extrica. It is interesting that there were single papers from these mentioned publishers and two papers from certain other sources inside this Other Sources category, underlining the variety and broadness of the literature base.

**Figure 3:**
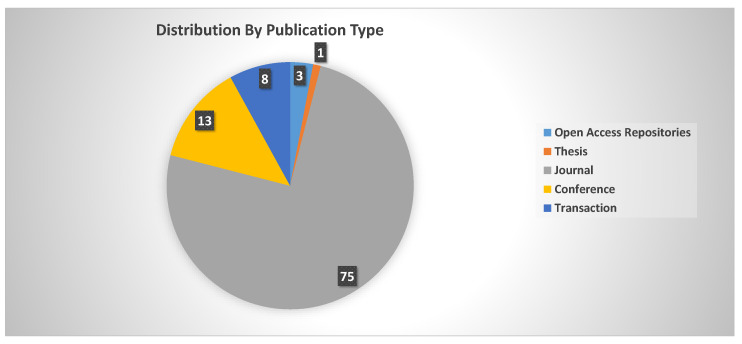
Distribution of papers by publication type.

Figure 3 provides a visual representation of the distribution of the reviewed papers among various publication categories, indicating the wide range of sources that were taken into consideration in this literature analysis. Out of the 114 papers reviewed, 75 papers were taken from journals. Meanwhile, 13 papers were taken from conferences, the second-largest category, and 8 papers were transaction papers. One Master’s thesis and three publications from open-access repositories were also included in the review, showing the importance of including academic thesis and publically available repositories in our comprehensive study.

## 3. Importance, Requirements, Challenges, Effect and Illustration of Vibration-Based Condition Monitoring for Heavy Machinery

### 3.1. Importance of Predictive Maintenance for Heavy Machinery

1. Reduced Downtime: Predictive maintenance provides a timely schedule of repairs or maintenance before a problem occurs. Machine failures can be decreased by resolving possible faults before they become serious issues, ensuring that activities continue smoothly and without unexpected failures [56].
2. Cost Savings: Addressing maintenance issues in advance can completely minimise the cost of repairs. Usually, fixing a minor problem as soon as possible is less expensive than repairing major damage caused by a complete breakdown [57].
3. Enhanced Safety: Predictive maintenance improves working conditions by reducing unexpected faults or failures. It improves with the prevention of failures that result from breakdowns of machinery, minimizing the risk of harm to workers [58].
4. Optimized Efficiency: Heavy machinery operating at its best leads to improved efficiency and production. Predictive maintenance makes sure that equipment performs at its best, reducing energy waste and improving productivity [59].
5. Extended Equipment Lifespan: Regular maintenance that uses predictive analytics may be helpful to extend the life of heavy machinery. Fixing issues as they arise helps to prevent cumulative damage, allowing machinery to operate more effectively for a longer period of time [60].
6. Data-Driven Insights: Collecting and analyzing information from machinery improves in analyzing patterns of use, wear and tear, and failure modes. This information may be used later to fine-tune intervals for maintenance as well as optimize machinery performance [61].

### 3.2. Requirements of Varying Heavy Machinery for Vibration-Based Condition Monitoring

Vibration-based condition monitoring is an efficient tool applied in a wide range of heavy machinery applications, but its precise requirements vary based on a number of variables [62]. Below as an explanation of how various heavy machinery uses variations in their requirements for vibration-based condition monitoring:

1. Industrial Manufacturing Machinery: For best operation, machinery utilized in the production process usually expects high accuracy and low vibration. Condition monitoring detects any minor variations that may have an impact on the quality of the product. While many industrial machinery work continuously, real-time monitoring is necessary for avoiding unexpected downtime and maintaining production [63]. These machines can often be made of complex systems, and monitoring improves the detection of problems in various interconnected components.
2. Construction and Earthmoving Equipment: Construction machinery is subjected to hard geographical and dangerous surroundings, leading to rapid wear and tear. Condition monitoring helps in predicting problems and properly scheduling maintenance. Construction equipment is impacted by a variety of loads and usage patterns. Monitoring allows us to see how these fluctuations affect the health of the machinery [64]. Vibrations might indicate possible problems that negatively impact safety. Timely monitoring plays a role in the prevention of accidents caused by equipment failures.
3. Mining Machinery: Mining machinery works in difficult environments exposed to debris, dust and extreme temperatures. Vibration monitoring is required to ensure that these devices can deal with a difficult environment. Equipment degradation can be increased by frequent heavy loads and high-impact operations [65]. Monitoring allows for the identification of problems produced by these operations. In mining operations, downtime is costly. Condition monitoring assists in predictive maintenance by decreasing unexpected breakdowns and optimising efficiency.
4. Transportation and Heavy Vehicles: Trucks, railways, and other heavy machinery have high usage and are constantly used. Monitoring vibrations is important for detecting problems before they cause breakdowns, providing timely repair, and preventing downtime. Monitoring improves the safety and stability of vehicle components by decreasing accidents caused by mechanical faults [66,67].
5. Energy and Power Generation: The equipment used in power generation machinery works constantly, and unplanned failures can cause heavy breakdowns. Vibration monitoring allows for the detection of faults early to prevent breakdowns and provide continuous power production [68].

### 3.3. Advantages of Vibration-Based Condition Monitoring

1. Early Fault Detection: It allows for the early detection of any potential defects within equipment or structures, which allows for preventive maintenance before a breakdown occurs [69]. This helps to avoid costly repairs and downtime.
2. Predictive Maintenance: Vibration signals can be used to detect possible issues and schedule maintenance plans more effectively. This method optimizes repairs while minimizing disturbance to operations [70].
3. Improved Safety: Vibration monitoring allows for the detection of machine faults, which increases the safety of employees by avoiding accidents carried by machinery that fails [69].
4. Cost Savings: Early detection and predictive maintenance decrease repair costs that minimise unplanned downtime, which leads to significant long-term cost savings [71].
5. Enhanced Equipment Lifespan: Actively fixing issues discovered by vibration analysis helps to increase the useful life of machinery and equipment [9], maximizing operational efficiency.
6. Real-time Monitoring: Continuous monitoring provides real-time analysis of equipment performance [72] as well as frequent alerts for any defects or deviations from standard operating conditions.
7. Comprehensive Insights: Vibration data provide particular insights about the condition of various components within machinery [50], allowing for improved maintenance operations.
8. Increased Efficiency: Vibration-based monitoring allows for maintaining equipment efficiency by focusing on possible issues before they become serious, making sure of continuous productivity [73].
9. Data-Driven Decision Making: Data collected by vibration monitoring helps data-driven decision-making processes, allowing for better scheduling and allocation of resources for maintenance activities [65].

### 3.4. Challenges in Vibration-Based Condition Monitoring for Heavy Machinery

Vibration-based condition monitoring is an efficient technique for measuring the health of machinery; some of the challenges are mentioned below:

1. Data Interpretation Complexity: Vibration data analysis involves knowledge and experience. Accurately interpreting the data to differentiate between normal vibrations and those that could be signs of a problem can be challenging. However, predictive maintenance addresses these problems using cutting-edge algorithms and machine learning [72]. These systems analyze previous vibration data to identify patterns associated with normal operation vs. patterns indicating problems.
2. Variability in Machinery: Different machines vibrate in different ways, and it takes an in-depth examination of each unique kind of equipment to fully understand these variations [32]. Predictive maintenance is customized by learning machine-specific vibration patterns, which allows for distinguishing between normal vibrations and anomalies across a wide range of equipment types.
3. Baseline Establishment: It is important to create the starting point for normal vibration patterns for every part of the equipment. To differentiate normal vibrations from possible defects, a significant amount of data must be collected and analysed. Predictive maintenance creates a starting point by collecting a vast amount of vibration data and groups regular patterns of each piece of equipment separately [74], which helps discriminate between normal vibrations and possible defects.
4. Environmental Interference: Vibration measurements might be affected by external elements like humidity, temperature changes, or adjacent machinery, which can cause problems with analysis and interpretation. Predictive maintenance reduces environmental interference [20] by combining sensor data with context-based data, then applying algorithms to filter out outside factors and identify accurate machinery vibration patterns for precise analysis.
5. Sensor Placement and Maintenance: It is essential to set up sensors on the machine properly. Unreliable data might be generated by inappropriate sensor placement or sensor malfunction. Predictive maintenance solves sensor installation and maintenance challenges by implementing [75] comprehensive sensor calibration, frequent checks, and strategic placement techniques to ensure accurate data gathering and reduce possible defects.
6. Data Volume and Management: A considerable amount of data are generated by continuous monitoring. Managing, storing, and processing these data can be difficult, requiring an efficient system and data management tools. Predictive maintenance handles the issue of handling huge amounts of monitoring data by introducing streamlined data storage systems and powerful processing tools that efficiently manage [76], store, and analyze data for meaningful insights.
7. Cost of Implementation: Choosing the vibration monitoring systems, buying specialized equipment, and training workers can be costly, especially for smaller organizations or those on limited budgets. One possibility for reducing the cost of implementing predictive maintenance, in particular for smaller organizations, is looking into scalable and modular solutions that allow for setup installation [20], beginning with important or high-risk equipment.
8. False Alarms and Over-Maintenance: Poor vibration data interpretation can result in false alarms, resulting in redundant maintenance or repairs that can be time consuming and costly. To prevent false alarms and over-maintenance [77] resulting from poor data interpretation, predictive maintenance improves its algorithms as time passes by taking input from maintenance activities.

### 3.5. Effect of Breakdown in Heavy Machinery on Operational Costs and Safety

The operational costs and safety of heavy machinery industries can be greatly affected by breakdowns and unplanned downtime. By fixing problems, predictive maintenance plays a key role in reducing these risks.

1. Operational Costs: Breakdowns usually require major fixes, including the replacement of components, labor expenses, and, in some cases, the use of specialized services. These expenses increase, especially if unexpected harm happens to other parts [78]. Lost productivity has a direct relation with downtime [79]. When machinery remains unused, project deadlines are missed, production goals are not met, and money is lost. Unexpected breakdowns may require urgent repairs, which could result in higher charges because services are pushed to reduce downtime.
2. Safety Concerns: When machinery breaks down, there may be significant safety hazards [80] for people, repair staff, and operators. Unexpected failures may result in problems, harm to others, or dangers to the environment. Equipment failures can result in incidents that put worker safety in danger and damage equipment in sectors like construction, mining, and transportation.

### 3.6. Illustrations of Vibration Response for Faulty Conditions

Vibration monitoring is useful to detect different issues in heavy machinery because they show different vibration patterns.

1. Imbalance: Vibration data can show peaks that are symmetrical at the rotational speed frequency, which indicates an imbalance. These peaks often have higher amplitude, particularly at a number of rotational speeds, indicating unbalanced mass distribution between the rotating components [81].
2. Misalignment: Misalignment issues frequently cause vibration spectra to show sideband peaks at the running speed frequency. Fluctuations in amplitude are found at various rotating speeds, indicating the existence of misaligned components [82].
3. Bearing Faults: Bearing faults occur in the form of spikes or impulses in vibration signals at particular bearing’s specific frequencies [83]. In addition, the rise in high-frequency content in the vibration data might suggest defects such as spalling, pitting or surface degradation.
4. Looseness: Vibration signals might show non-synchronous peaks at different frequencies when there is looseness, which denotes loose connections [84]. There may be inconsistencies in the amplitude, particularly when loads or operating circumstances change.
5. Gearbox Faults: Faults in gearboxes commonly result in harmonic peaks at gear mesh frequencies [74]. Gear mesh frequency is a product of the total amount of teeth on the gear multiplied by its running speed. These peaks may look twisted or vary in amplitude, indicating gear wear or damage.
6. Components: Cracked components might cause temporary impulses or spikes in the vibration data that appear periodically [85]. Cracks or fractures can also generate special changes in frequency components.
7. Soft Foot: Soft foot situation may result in unbalanced peaks at the running speed frequency in vibration data. The stability may be shown by the intensity of these peaks changing according to variations in load or operating conditions [86].

## 4. Review of Sensors, Simulations and Datasets for Vibration-Based Condition Monitoring

Heavy machinery in operation generates vibration data as a result of various underlying mechanical and operational factors. These components can both be expected results of machinery operation and possible problems that need to be monitored over time and handled. Various approaches have been used in the field of heavy machinery condition monitoring to collect important vibrational data. In particular, these methods include the use of accelerometers, techniques based on simulation, and even research studies carried out with data obtained from some repositories. The variety of data collection techniques brings out the complex character of research in this area with each method providing distinct insights and benefits. This in-depth examination of the literature shows researchers’ various techniques to gather the essential vibrational data required for their studies.

### 4.1. Accelerometer

An accelerometer is a sensor that measures acceleration. It is widely used in electronic gadgets including cell phones, fitness trackers, and automobile systems. The second rule of motion, which states that the force acting on an object is equal to the mass of the object multiplied by its acceleration (F = ma), is the basis upon which accelerometers work. Accelerometers work on the inertia principle. They analyze the force generated on a mass when it is accelerated. The mass within the accelerometer opposes changes in motion, and the force it feels is proportional to the applied acceleration. There may be different types of accelerometers. Our literature review of the selected papers shows that researchers used a diverse range of accelerometer types in their experiments. Piezoelectric uniaxial accelerometers, PCB piezoelectric accelerometers, ADXL335 and ADXL345 accelerometers, MEMS dual-channel accelerometers, and others were explicitly specified in multiple studies. It should be noted, that some papers give specific information about the accelerometer types used. The variety of accelerometer possibilities, extending from uniaxial to triaxial (that measures vibration along one axes and three axes, respectively), wired to wireless, and many branded models, highlights these sensors’ versatility and adaptability in addressing the individual needs of diverse research and application scenarios. A total of 30 people, Mao et al. [34], Wu et al. [87], Koutsoupakis et al. [5], Li et al. [88], Ong et al. [45], Elvira-Ortiz et al. [25], Wang et al. [89], Feng et al. [35], Kannan et al. [90], Civera et al. [91], SUN et al. [92], Sharma et al. [29], Inturi et al. [93], Xu et al. [36], Mystkowski et al. [8], Mukherjee et al. [94], Naveen Venkatesh et al. [3], Bhowmik et al. [19], Mazzoleni et al. [95], Rafiq et al. [96], Hu et al. [97], Mukherjee et al. [98], Fernando et al. [99], Lin et al. [100], Krot et al. [101], Sajedi et al. [102], Demircan et al. [103], and Sepulveda et al. [104] used accelerometers as part of their data gathering used in their research. Additionally, these research studies did not provide detailed information about the brand or model of the accelerometers used. Uniaxial accelerometers were used by Yaghoubi Nasrabadi et al. [105], Gómez et al. [106], and Vila-Chã et al. [107], while Yang et al. [108] used a Triaxial Accelerometer.

#### 4.1.1. Piezoelectric Accelerometer

A piezoelectric accelerometer uses the piezoelectric effect, which causes certain materials to produce electrical charge when they undergo mechanical stress (vibration). The amount of charge is directly proportional to the applied force. Balachandar et al. [13], Mongia et al. [27], Pranesh et al. [109], Patil et al. [39], Harish et al. [110], and Joshuva et al. [111] used the piezoelectric accelerometer. In contrast, Vila-Chã et al. [107] used a piezoelectric uniaxial accelerometer. A piezoelectric uniaxial accelerometer is like a piezoelectric accelerometer but is designed specifically to measure vibrations in a single direction. Endevco 751-10 is a lightweight piezoelectric accelerometer used by Michalak et al. [112]. This piezoelectric accelerometer is lightweight, inexpensive, and has built-in electronics. It was created especially to measure vibration on small structures.

#### 4.1.2. PCB Piezoelectric Accelerometer

The PCB piezoelectric accelerometer is an accelerometer made by PCB Piezotronics that measures vibrations using transduction techniques including piezoelectric, piezoresistive, and capacitive. PCB piezoelectric accelerometers were used by Morgan et al. [113]. Dabrowski et al. [114] used the PCB 356A15 accelerometer, which is a high-temperature sensor. Sun et al. [115] used a PCB 356A16-K accelerometer, which is a triaxial and high-sensitivity accelerometer. The Accelerometer sensor PCB 356A16-K was used by Sun et al. [115].

#### 4.1.3. Wired and Wireless Accelerometer

Wired and wireless refers to the communication mechanism (wired or wireless)—not the fundamental accelerometer technology. Both wired and wireless accelerometers include a variety of sensing methods, including piezoelectric and MEMS. Zanelli et al. [116] and Wang et al. [117] made use of wired and wireless accelerometers.

#### 4.1.4. Acoustic Emission Transducers

Acoustic emission transducers capture and analyze acoustic signals released by materials during stress or deformation. They monitor oscillations indirectly by generating acoustic waves. Marticorena et al. [38] used acoustic emission transducers for recording vibration data.

#### 4.1.5. MEMS Accelerometer

MEMS (Micro-Electro-Mechanical Systems) accelerometers are small-scale vibration sensors produced via micro-fabrication processes. MEMS accelerometers have been observed in work by Ompusunggu et al. [118], Zonzini et al. [119], and Patange et al. [120], and a MEMS dual-channel accelerometer was studied by Sharma et al. [121]. MEMS dual-channel accelerometers are similar to MEMS accelerometers but monitor vibrations along two axes.

#### 4.1.6. Stud-Mounted Single-Axial Accelerometer

It uses piezoelectric or piezoresistive sensors to record acceleration along a single axis. Stud-mounted single-axial Accelerometers were found in work completed by Chesnes et al. [37].

#### 4.1.7. Magnetic Tri-Axial Accelerometer

It simultaneously measures acceleration along three axes using magnetic sensing technology. Chesnes et al. [37] made the use of magnetic tri-axial accelerometer.

#### 4.1.8. CTC Accelerometer

Capacitive transduction accelerometers, or CTC accelerometers for short, monitor acceleration by using changes in capacitance. These exact accelerometer types were chosen based on their usefulness in the respective studies. Lu et al. [122] also used the CTC accelerometer.

The accuracy of data recorded by these accelerometers is summarized in Table 1 below.

Simulations and existing datasets play an essential role in improving vibration-based condition monitoring studies with their controlled environments and real-world data; however, the impact they have is limited by their ability to mimic complex real-world variability and conditions precisely. It has some advantages as well as some limitations, as given below.

### 4.2. Advantages of Simulations and Existing Datasets

1. Cost-Effectiveness: When compared to practical realities, testing, simulations and existing datasets data can be more cost-effective. They allow the creation of virtual models that simulate equipment behavior under different conditions [123], helping researchers examine many different situations without developing the cost of real testing.
2. Data Generation: Simulations create vast quantities of data, allowing for an in-depth understanding of how machinery performs under various loads, speeds, and temperatures. Such data and existing datasets data can be beneficial for training machine learning models for use in condition monitoring [124].
3. Risk-Free Testing: Simulations minimize the risk associated with in-person testing, mainly when working with costly or significant machinery. It allows testing without the risk of damage or hazard [125].
4. Predictive Analysis: Based on sequences identified by vibration data, existing datasets data, as well as simulations, provide predictive analysis, allowing experts to predict probable failures or degradation in machinery performance.
5. Training and Education: Simulations and existing datasets data serve as an environment for training operators and maintenance engineers to identify different vibration patterns and to understand their importance in condition monitoring.

### 4.3. Limitations of Simulations and Datasets in Condition Monitoring

1. Model Accuracy: The models’ precision has an important effect on simulation accuracy. Existing datasets data and simulations might not accurately represent the complexity and specifics of real-world machinery activities [126], leading to possible predicted failures.
2. Limited Real-Time Data: Although simulations generate huge amounts of data, still datasets and simulations could fail to include every aspect of real-time situations and scenarios observed by machinery in the field [127]. This can lead to misunderstandings about real operational unpredictable conditions.
3. Dataset Generalization: It is possible that some failure modes or behaviors of machinery are not fully covered by existing datasets and sometimes cannot be simulated. This limitation could make creating effective condition monitoring systems more difficult, especially for new or unexpected issues.
4. Validation Challenges: Verifying simulation and dataset data results using real-world data is important. Variations in these results during the actual conditions could be due to the unaccounted factors or simulation model faults [128].
5. Complexity and Resources: Producing and upholding accurate simulations requires vast knowledge, time, and computational resources. These resources may not be available to all organizations.

In the review of 114 research papers, some studies use simulation-based gathering methods. In several cases, researchers used software to create realistic environments that resembled real-life situations but were explicitly artificial. They carefully collected vibration data from heavy machinery within these virtual environments and processed it for condition monitoring. The innovative part of these studies was the practical application of simulation software to reproduce complex real-world situations, allowing researchers to investigate and interpret vibration patterns in controllable and accurate settings. The given below Table 2 shows the simulation software used by researchers for simulating the scenario for collecting vibration data for performing condition monitoring.

A subgroup of 13 studies from the same group of 114 papers took a different approach to their studies by choosing to use preexisting datasets. These datasets, given in Table 3, have been obtained from various sources, including open-access repositories and contributions from other research community members. Researchers could pick up their studies into the dynamics of heavy machinery vibrations by using these preexisting data sources. In addition to saving time and money, this approach facilitated collaboration by making essential data accessible for research.

## 5. Review of Heavy Machinery and Their Equipments

**Figure 4:**
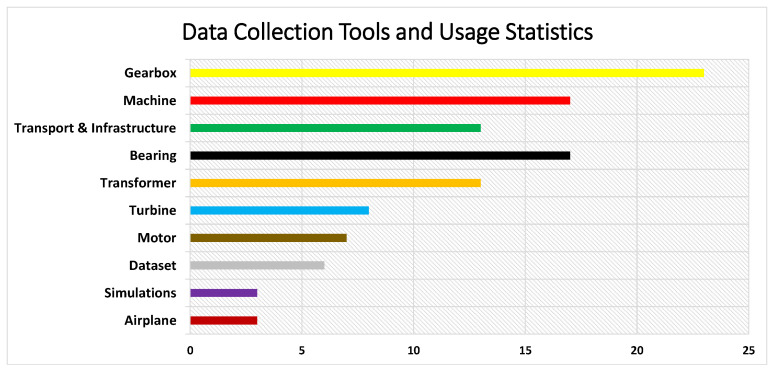
Data collection devices in research papers.

We reviewed 114 papers, but only 101 research articles mention the data collection process. The discrepancy is caused by the fact that several of the papers under consideration focused on activities like simulations or processing already existing datasets that did not directly involve the usage of machines. In addition, some 101 papers that used machines still mentioned that they performed their model on simulations or datasets alongside the machines for their research. The different kinds of tools and machines used in the reviewed papers differ. We classified them based on features and characteristics they had in common. Figure 4 depicts how data-collecting sources are spread across the 101 studies we examined. Each bar represents a specific type of data-collecting source.

The most extensive bar on the chart indicated in the above Figure 4 belongs to Gearboxes, which were the primary data-gathering source in 23 papers. Bearings and Machines come in second and third, with 17 publications each, showing a strong interest in these fields of study. With eight papers, the categories Transformers and Turbines are particularly prominent. Turbines, Datasets, Motors, Airplanes, and Simulations, on the other hand, have considerably smaller bars, each representing eight, six, seven, three, and three papers, respectively.

### 5.1. Gearbox

The gearbox transfers power from the engine to the wheels via a series of gears that may be moved to change the speed and torque. The gears are set in various configurations to provide multiple speeds while retaining engine/motor performance. A gearbox comprises an input shaft linked to a power source, often an engine or electric motor, and an output shaft coupled to the device or mechanism that requires power. The gearbox’s component is a collection of gears, which are toothed wheels of varied diameters that mesh together. A gearbox’s can be adjusted by switching out its gears. Low gear is perfect for power-demanding jobs like starting a big vehicle or climbing hills because it has a larger output gear and a smaller input gear, which increases torque while decreasing rotational speed. On the other hand, high gear is better suited for reaching higher speeds since it has a smaller output gear and a larger input gear, which results in more acceleration and less torque. Higher driving speeds are also possible using a high-speed gear configuration, which is likewise made up of a larger input gear and a smaller output gear. Some gearboxes also allow us to reverse the rotational direction. Furthermore, there is a neutral mode in which no gears are engaged, allowing the engine to run without passing power to the output. In short, the flexibility and capacity to fine-tune the power distribution of a gearbox make it an essential part of a wide range of equipment and vehicles, maximizing their performance for various activities and conditions. The literature review encountered various gearbox subtypes used in different research papers. In some papers, they mentioned the specific type of gearbox, while in others, they just mentioned that they had collected data from the gearboxes without mentioning their particular type.

#### 5.1.1. Single-Stage Gearbox

A single-stage gearbox has only one gear to change the torque or speed. Mao et al. [34] collected vibration data from a single-stage gearbox.

#### 5.1.2. Planetary Gearbox

A planetary gearbox is a type of gearbox in which the output and input have the same axis of rotation. Koutsoupakis et al. [2], Dabrowski et al. [114], Sharma et al. [29], and Lipinski et al. [140] collected vibration data from the planetary gearbox.

#### 5.1.3. Two-Stage Gearbox

A two-stage gearbox system has two sets of gears, giving two different combinations of gear ratios for mechanical systems with varied speed and torque. Koutsoupakis et al. [2] used a two-stage gearbox as a data collection device.

Elvira-Ortiz et al. [25] and Zhao et al. [141] collected data from an electrical motor gearbox that joins an electric motor to a mechanical system and uses gears to regulate the motion’s torque and direction. Vos et al. [133] and Mauricio et al. [142] acquired data from helicopter gearboxes to monitor these systems’ conditions. Data from a rolling mill gearbox was used by Lu et al. [122], data from a blender motor gearbox was collected by Mukherjee et al. [94], and data from a wind turbine gearbox was used by Amin et al. [41]. Some articles, such as those by Ong et al. [45], Peters et al. [143], Wang et al. [134], Civera et al. [91], Inturi et al. [93], Mazzoleni et al. [95], Samant et al. [4], and Krot et al. [101] did not mention the gearbox type. Data from a ring gear was used by Hu et al. [97], while Xu et al. [36] concentrated on gearbox test data. Feng et al. [35] recorded data from a spur gearbox.

### 5.2. Bearing

To reduce friction and enable the smooth movement or rotation of elements within machinery, bearings are critical mechanical components. Bearings comprise an outer, stationary ring inside the machine’s structure and an inner ring coupled to a rotating shaft or component. They operate on the fundamental idea of decreasing friction. Rolling components, such as balls, cylindrical rollers, or tapered rollers, are positioned between these rings. These rolling components are essential because they allow movement with minimal resistance. When an external force is applied to rotate or move the inner ring, the rolling parts help by rolling, rather than sliding, between the rings. This design reduces friction, resulting in less wear and energy loss. Bearings are classified into several varieties, including ball bearings, roller bearings, thrust bearings, and plain bearings, each designed for specific uses. Several papers in our review collected vibration data from bearings or bearing-like components. Wang et al. [89] acquired vibration data from wheel rails, which have qualities similar to bearings as rotating components. Although some papers did not specify the exact bearing type, some did. Amin et al. [16], Brusa et al. [136], Samant et al. [4], Patil et al. [39], Patil et al. [131], and Sepulveda et al. [104] indicated collecting data from bearings but did not specify the type of bearing. Marticorena et al. [38] focused on bearing cage data, Nirwan et al. [144] focused on rolling mill roller bearings, and Cornel et al. [6] and Aburakhia et al. [11] focused on roller bearings. Data were obtained by Espinoza-Sepulveda et al. [145] from a rotating rig, by Li et al. [146] from a ball screw, by Sun et al. [115] from a throw rod, by Gurusamy et al. [147] from a stator, by Mukherjee et al. [98] from computer cooling fans, and by Mystkowski et al. [8] from a hay rotatory tedder.

### 5.3. Turbine

A turbine is a mechanical tool that transforms fluid energy, usually from steam, gas, or water, into mechanical energy that can be used to produce electricity. High-pressure fluid is pumped into blades installed on a rotor in a way that fits with the fluid dynamics and thermodynamics rules that control turbine operation. In the context of heavy machinery condition monitoring, we reviewed works on wind turbine condition monitoring, particularly those linked to electricity production. Amin et al. [16] and Rafiq et al. [96] collected the vibration data from wind turbines but did not confirm the component within the turbine. Yaghoubi et al. [148], Yaghoubi Nasrabadi et al. [105], and Joshuva et al. [111] gathered data from turbine blades, while Murgia et al. [30] used data from an onshore wind farm.

### 5.4. Transportation and Infrastructure

We examined papers on condition monitoring using vibration data for bridges, railway lines, and a historical building under the area on transportation and infrastructure. The study of several bridge types, including steel truss bridges, X24 bridges, concrete bridges, concrete culvert bridges, and steel beams, was featured in some papers, including Sharma et al. [121], Zonzini et al. [137], Fernando et al. [99], Lin et al. [100], Nie et al. [132], and Zonzini et al. [119], respectively. Vila-Chã et al. [107] focused on a historic building. Nowakowski et al. [149] focused on the railway tracks, and Zanelli et al. [116] addressed the condition monitoring of railway line mileage. Milosevic et al. [150] investigated railway geometry, Gómez et al. [106] gathered data from railway axles, and Naveen Venkatesh et al. [3] gathered data from the tool holder. To show the use of simulation data in railway condition monitoring studies, Tsunashima et al. [12] also collected data while simulating a railway track. In this section, a range of infrastructure components were covered.

### 5.5. Electric Motors

Several papers in our review used motor vibration data to perform condition monitoring. Data were collected from electric motors by Koutsoupakis et al. [2], Li et al. [88], and Espinoza Sepúlveda et al. [151]; from industrial electric motors by Ompusunggu et al. [118]; from Kirloskar motors by Raghavendra Pai et al. [152], pumps by Demircan et al. [103], and single-phase induction motors by Rahman et al. [42]. The given below Table 4 shows the type of machine used for data collection in which article. Wu et al. [87] and Mauricio et al. [142], in particular, obtained data from transformers to aid in condition monitoring. In contrast, Rajapaksha et al. [153] focused on power transformer windings for their data collection. Particularly, Yin et al. [135] and Serin et al. [85] examined drones and gathered vibration data for condition monitoring. In contrast, Aburakhia et al. [11] extended the focus to incorporate the transmission system for helicopters. Balshaw et al. [1], Elvira-Ortiz et al. [25], Tsunashima et al. [12], Chesnes et al. [37], Priebe et al. [40] and Harish et al. [110] are among those studies who prefer to use existing datasets as the basis for their research. Similar to this, for condition monitoring, Skowronek et al. [154], Jombo et al. [20], and Hu et al. [155] decided to use simulation-based techniques. These unique data collection techniques highlight the diversity of methodologies used in the condition monitoring field, enabling an in-depth review of the problem.

## 6. Summary

Heavy machinery condition monitoring is essential in industrial operations, ensuring optimal functionality, preventive maintenance, and risk mitigation. This section discusses research efforts identified by different methods and tools for collecting data. The initial part investigates experiments that used various devices to capture vibration data. It then moves to studies that use existing datasets, illustrating the effectiveness of existing data repositories. Finally, it continues through research studies that explore simulation techniques, providing prediction models to mimic and analyse machinery behavior.

### 6.1. Accelerometer

#### 6.1.1. Gearbox

Mao et al. [34] used an accelerometer to record vibration data during a lubricated gear test containing silica sand. The experiment attempted to look into the evolution of wear on gear teeth by examining initial abrasion and pitting caused by sand addition. Through analysis and comparison with absolute transmission error (TE) measurements, the vibrations recorded by the accelerometer were able to shed light on the mechanisms and degree of wear in the gear teeth. At Amirkabir University of Technology, Ong et al. [45] collected vibration signals from the gearbox. They created an experimental setup that had four main parts: a disc brake system that controlled rotational speed, a gearbox, a three-phase electromotor that powered it at a minimum speed of 1420 RPM, and various electronic components. An accelerometer with a 10 kHz sampling rate fixed on the gearbox frame recorded the vibration data. Ten seconds were given for collecting data for three different health conditions: worn, chipped, and normal teeth. Sharma et al. [29] used an electromechanical system (EMS) with a parallel gearbox (PGB) acting as a load machine on an induction motor (IM) in their experiment. Several faults—chipped sun gear, pitted planet gear, and combination faults—were induced on the gears using an end milling machine. The experimental data show the severity levels of the faults at various rotational speeds (35, 40, 45, and 50 Hz). The process is divided into steps: First, for all operational situations, electrical current signals from the IM phase are captured during no-load conditions at a sample frequency of 12,800 kHz for approximately 5.2 s. The obtained signals are then predicted using autoregressive (AR) modelling. The residual signals, derived by subtracting the AR model-predicted signals from the originals, are used for entropy-based feature extraction using the wtMFDE approach with a scale factor of 5. The fluctuation of these features with the degree of damage in the PGB system is investigated to comprehend their response to various health situations. Inturi et al. [93] attached 1 HP 1500 rpm AC motor to the gearbox’s input high-speed stage (HSS) shaft to power a three-stage spur gearbox in the experimental setup. The motor speed is managed by a variable frequency drive communicating with a computer through a data-gathering device. Heavily lubricating is achieved using heavy gear oil. This study focuses on the HSS components because of their greater working speeds, which make them crucial. Thirteen gearbox health conditions are considered, ranging from different degrees of inner and outer race flaws to broken pinion tooth defects. An accelerometer on the HSS bearing housing is used to gather vibration signatures, while a microphone placed above the bearing housing is used to record audio signals. For every 200 observations in each health state category, 8192 points are collected at a sample frequency of 16 kHz. The Hurst exponent is calculated for additional analysis using raw time-series vibration and acoustic signals. Xu et al. [36] used two tests by the model to verify its primary focus on bearing and gear systems: a bearing rig for clearance impact and a gearbox rig for system vibrations. The bearing rig’s clearances exhibit different amplitudes at BPFO, which was verified by other studies. An accelerometer collected vibrations from both good and defective gears in the gearbox, and tests showed distinct features that validated the model’s accuracy. An IMU sensor and current/voltage sensors were used in the vibration monitoring system by Mukherjee et al. [94], and data were sent to an Arduino-based ESP-32 board. A Raspberry Pi gateway gathers and analyzes these data using MQTT for reliability. The Raspberry Pi, a laptop, and the Microsoft Azure cloud are all used in the tests. Two operational states (empty and filled with water) are acquired using a consumer-grade mixer blender and separated for training and testing. The trained model is then used on various machines to identify operating states, which present issues due to equipment variation and the utilization of low-cost components. Mazzoleni et al. [95] focused on a steel mill that produces different billet profiles, analyzing the last cage’s gearbox in the rolling mill line, which plays an important role due to high stress. Vibration signals from accelerometers, oil pressure, temperature, motor current, RPM, and torque were all recorded at different frequencies. Data were collected for every ten billets, producing a set of rows and columns corresponding to the sensor numbers. A maximum 10 kHz sampling frequency used for 15 s for each billet resulted in a memory burden of roughly 100 MB. During the September 2019–October 2020 observation period, one failure resulted in the rolling bearings in cage 18 breaking down and releasing smoke and odors. Industrial ICP accelerometers (PCB 603CX1) were permanently placed on the gearbox’s input shaft bearing supports by Krot et al. [101], recording vibration data at a sampling frequency of 2 kHz. The torsional load measurements from the motor shaft and angular clearance checks in the gearbox were also collected. The analysis showed a high frequency of maintenance requirements, especially for bolt stud loosening in locations with significant shaft movement. The torque and vibration tests showed that the dynamic response of the shaft varied with rotational direction with the highest vibration happening where opposite forces acted on the shaft. Dabrowski et al. [114] performed an experiment in the Laboratory of Mechanical Diagnostics at AGH University of Science and Technology in Krakow, Poland, and concentrated on the planetary gearbox. A Laser Tacho Probe MM0360 was used to record the rotational speed of the gearbox input shaft, and a PCB 356A15 accelerometer was used to record vibration signals on the gearbox case. Misalignment, imbalance, and the combination of both were some defects that were studied. By adding mass to the coupling, the imbalance was created on the input shaft, and misalignment was created by moving the motor support. Ompusunggu et al. [118] compared low-cost MEMS accelerometers (ADXL001-70 and ADXL1002) to a high-end PCB355 B12 accelerometer on a gearbox configuration for fault injections. While the gearbox was stationary, data were collected from the sensors (50 kHz sampling), revealing higher noise density in the ADXL001-70. The second test used a faulty bearing, and vibration data from the accelerometers showed an inner race fault at 54.3 Hz in the envelope spectra at 2100 rpm (35 Hz). Hu et al. [97] gathered vibration data from accelerometers and a speed sensor at a high sample rate in the study of wind turbine gearbox conditions. Damage to the second-stage ring-gear tooth was found during an inspection. A study was performed using accelerometer data, which showed blurring effects caused by variable-speed operations, making diagnosing gear degradation difficult. A synchronous-analysis technique with instantaneous shaft speed determination was used to address this. The second harmonic of the high-speed gear meshing was identified as a speed-tracking index by analyzing the vibration signal using a proposed approach based on Short-Time Fourier Transform (STFT), which aligned well with the gearbox’s high-speed shaft (HSS) reference speed. When vibration signals were resampled based on the second-planetary-stage input shaft, synchronous averaging clearly showed gear-meshing orders and harmonics associated with ring-gear breakage, as indicated by the planetary gear set phenomena. Six high-rolling mills were formed up the Universal Crown Control Mill (UCM) cold rolling line in the work of Lu et al. [122]. Accelerometers were used to record roll vibrations in real time. To detect vertical roll movements, four CTC accelerometers were installed in response to chatter, which was noticed during the high-speed processing of thin strips.

#### 6.1.2. Bearings

Sepulveda et al. [104] collected vibration data from many components in an experimental rotating rig, including steel shafts coupled by a rigid coupling and supported by four ball bearings. One shaft is connected to a three-phase electric motor via a flexible coupling and holds two balancing discs, while the other holds one. The inherent frequencies of the rig have been measured and correlated with specific mode shapes, making it easier to identify vibration issues. The data collected from accelerometers at various bearing cases provide information into the rig’s health at different rotor speeds, including healthy and faulty states. At every bearing housing, these accelerometers with a sensitivity of 100 mV/g and a frequency range of up to 10 kHz have been carefully placed at 45-degree angles from vertical and horizontal directions. Marticorena et al. [38] developed an REB test rig for bearings with an outer diameter of up to 160 mm that was used in the experimental evaluation of the approach. A three-phase electric motor with a variable frequency drive was used for shaft speed control. Weights and a lever mechanism were used to load the machine. A 256-point incremental encoder on the shaft, a 4371 accelerometer on the test housing, a piezoelectric transducer for high-frequency stress waves, and a cage sensor to monitor cage rotation were all the critical sensors that were used. The customized cage sensor uses a permanent magnet and a magnetoresistive transducer to detect movement in the cage. The high-frequency piezoelectric sensor, coupled to a preamplifier and a passband filter, was carefully placed near the bearing’s load zone for optimal detection of stress waves. A 15 kHz low-pass filter was used to filter the accelerometer signal. The tests were carried out with a specific 6209 ball bearing using stamped sheet steel cages that were lubricated with an automotive transmission fluid (Castrol ATF Multivehicle) that was defined by its kinematic viscosity, density, and pressure-viscosity coefficient. The ZDJ9 railway point machine, an essential part of China’s railway network, was used by Sun et al. [115] in the experimental setup carried out at Xi’an Railway Signal Co., Ltd. (Xi’an, China). The PCB 356A16 K accelerometer sensor was used to collect vibration signals, which were also sampled at 5.12 kHz. Eight fault conditions were simulated (created in reality), and the related vibration signals were recorded.

#### 6.1.3. Turbine

The vibration data used by Rafiq et al. [96] were obtained from the National Renewable Energy Laboratory’s Wind Turbine Gearbox Vibration Benchmark. The signals were recorded using the PXI-4472B National Instruments DAQ system at a high speed of 40 kHz per channel, utilizing eight accelerometers mounted throughout the gearbox. The dataset contains ten 1-min segments recorded under specific operating conditions: 1800 RPM on the high-speed shaft, 50% rated power, and 22.1 RPM on the main shaft. The gearbox design includes a planetary gearbox stage and two parallel stages (intermediate and high speed). Joshua et al. [111] identified and determined possible defects in wind turbine blades by analysing their condition. The tests were carried out on a 50 W, 12 V variable wind turbine positioned on a fixed steel stand in front of an open circuit wind tunnel outlet. Wind speeds ranging from 5 to 15 m per second were used to simulate various environmental situations. A piezoelectric accelerometer (DYTRAN 3055B1) is positioned near the wind turbine hub and connected to a DAQ system (NI USB 4432 model) via a wire for collecting vibration signals. The turbine was initially fault-free, and vibration signals were recorded with several parameters: a sample length of 10,000, a sampling frequency of 12 kHz, and a minimum of 100 samples for each condition, which were all monitored with NI LabVIEW. The turbine was set to run at 850 rpm, and vibrations were identified vertically at the hub using an accelerometer. Following that, different flaws, such as blade bend, crack, erosion, loose contact at the hub, and blade pitch angle twist, were simulated one at a time on individual blades while keeping other blades in good condition. Each fault state generated various vibration signatures that were captured and analyzed to determine fault diagnosis and characterization. Nasrabadi et al. [105] covered vibration response analysis on Equiax Polycrystalline Nickel alloy first-stage turbine blades. Using a Single-Input Multiple-Output (SIMO) configuration, including a single actuator and a pair of sensors, frequency response function and amplitudes were collected for every blade. The data were collected from 150 healthy and 79 defective blades, covering many damages such as overheating, airfoil cracking, corrosion, thin walls from casting, and service wear. Civera et al. [91] proposed an entropy-based condition monitoring technique that was confirmed using experimental recordings from an onshore wind farm in northern Sweden, focusing on one of eighteen monitored turbines. The damage that occurred to the output shaft bearing as a result of an inner raceway failure requires the replacement of the gearbox after two years of service. The accelerometer data from the damaged turbine’s bearing were combined to generate two signals that simulated non-stationary structural circumstances. Signal 1 consisted of pre and post-replacement segments used for parameter setting discussions and damage index testing. Signal 2 contained several tracts before and after the damage, demonstrating the approach’s versatility with varying rotating speeds. Fast Fourier Transform (FFT) analysis was performed on standardized accelerometer data, exposing difficulty in frequency-based damage diagnosis due to various harmonic components post-replacement, indicating limits in typical FFT-based approaches for wind turbine gearbox bearing failures.

#### 6.1.4. Transport and Infrastructure

Naveen Venkatesh et al. [3] used an ACE Micromatic-Classic 20T CNC turning machine, a piezoelectric accelerometer, and a signal conditioning machine as part of their system. The accelerometer was used to gather data from a mild steel shaft, and these signals were digitized for computer storage using a DACTRAN FFT analyzer. They monitored tool conditions by manually inducing defects in the tooltip and capturing vibration data with adhesive-mounted accelerometers. Various fault situations were simulated, including low and high bluntness and a loose tooltip. A pretrained network was used to handle the vibration data for condition monitoring. For signal acquisition, sampling parameters were adjusted to a frequency of 24 kHz. After a minute of system stabilization, 100 signals were captured for each fault scenario during rotating operations. Zanelli et al. [116] fitted a hybrid monitoring system consisting of wireless sensor nodes, a gateway and an axle box generator during a three-month field test on the Verona–Rotterdam railway track. The system was created to collect data on wagons while they were in operation. Data from wireless nodes were compared with data from a single wired accelerometer. These nodes, which were positioned carefully on the wagon frame and central bogie, connected wirelessly to a gateway, which gathered, processed, and sent data to the Cloud. The gateway monitored data collection and used a power-saving logic to turn on or off the PC based on train movement. It was powered by a rechargeable battery through the axle-box generator. With this setup, sensor data could be automatically acquired when the train moved and linked to GPS. For structural monitoring, a sensor network architecture made up of PZT discs, MEMS accelerometers, and a gateway (GW) was created by Zonzini et al. [119]. For reliable communication in electrically noisy environments, a wired multidrop sensor area network (SAN) bus using RS-485 was used in the setup. Continuous, high-speed data acquisition from structures was made possible by data-over-power communication. The system supports up to 12 channels, collecting data at 1 ks/s and sending it sequentially via lossless encoding. A three-way handshake protocol was used for synchronizing nodes, bringing accurate data together and providing a comparison for post-processing. Sharma et al. [121] tested cutting joins on a steel truss bridge model to cause damage. There were three types of damage scenarios introduced: single, double, and triple damage cases. A 5 kg hammer used to create multiple strikes across the bridge span. MEMS dual-channel accelerometers, which recorded voltage outputs in proportion to accelerations, had been placed at different nodes for vibration measurements at 200 samples per second. The sensors have been carefully positioned at nodes where inclined and vertical members interact with one another. The vibrational responses were measured and analyzed at 18 different locations. A system that filtered and stored signals in ASCII format helped in data acquisition. Gómez et al. [106] used a specialized testing rig to collect the vibration response data for wheelsets from Dannobat Railway Systems. With the help of hydraulic actuators and a driving system, this rig allowed for controlled loading on a bogie structure. Six uniaxial acceleration sensors have been carefully positioned on each wheelset to record vibrations in both the radial and axial directions. The measurements, which took about 30 min each, produced a large dataset with around 960 signals produced per vibration direction for each crack level evaluated across the four wheelsets. This setup enabled an in-depth analysis of vibration responses in steady-state conditions. The objective of the work by Vila-Chã et al. [107] was to develop a baseline of dynamic features for implementing a condition monitoring technique at a cloister’s south wing. Piezoelectric uniaxial accelerometers with high sensitivity were used in five different spatial arrangements with a total of forty degrees of freedom distributed among the highest and intermediate levels. Three four-channel data acquisition modules and a four-slot USB chassis was used to record the data, providing a 200 Hz sample rate for 20-min. intervals in order to continuously record a system’s main period. These tests were carried out during museum operations to avoid downtime, although they did interrupt cloister access. The deployment of cabled sensors and extension cords was relatively intrusive, with sensors located approximately 30–40 m from the acquisition system, connecting floors via inner court windows. Patil et al. [131] used the measurement of the mechanical responses caused during regular service situations. Field vibration measurements were made with Guralp Fortis accelerometers set up to properly record the first two mode shapes. The measurements were taken with a data logger, which recorded the acceleration–time history information generated by passing automobiles. When automobiles entered the bridge, the accelerometers were triggered, collecting vibrations even after the vehicles left, which is a phenomenon known as free vibration. Bridge 43/10 measurements were taken in 2017; Bridge 13/3 measurements were taken in 2017 and 2018. Fernando et al. [99] determined the modes of transportation features of two bridges; their methodology consists of experimental and numerical analysis. The Eigensystem Realization Algorithm was used to analyze acceleration data and measure structural responses during the experiment. Eigenvalue analysis of finite element models using SAP2000 software allows for the numerical evaluation of dynamic features. Two Sri Lankan single-span PC bridges, 43/10 and 13/3, were chosen for this study. A data logger was connected to sensors which were placed carefully to record mode forms and vibrations caused by passing cars. With wired accelerometers sampling at 200 Hz, vibration examinations were made under normal operating conditions. Vehicle-induced vibrations have been recorded, including free vibrations that were maintained after cars had left the bridge. Since June 2016, the Governor Macquarie Drive (GMD) bridge in Liverpool, New South Wales, Australia, has had an SHM system with strain gauges and accelerometers. In 2018, the bridge was expanded from two to three lanes, increasing its width and sensors. From July 2016 to February 2017, 22 strain gauges, 12 accelerometers, and 1 thermocouple have been used by Lin et al. [100] for continuous data collection. Due to the increased stiffness of GMD, the observed acceleration responses were significantly less than those of larger bridges. Different truck speeds (20 to 60 km/h) generated different strain and acceleration responses with greater speeds producing sharper strain responses. FDD was applied to the acceleration responses to determine the natural frequencies before and after increasing, yielding frequencies of 16.11 and 12.89 Hz, respectively. These data helped in understanding the bridge’s structural behavior under various traffic situations, considering the bridge’s sensors, traffic loads, and operational modal analysis approaches.

#### 6.1.5. Motors

Wu et al. [87] developed a MECM (Mechanical Equivalent Circuit Model) for transformer winding and investigated its use in vibration-based condition monitoring. The study shows that the MECM accurately represents the vibration features under spacer nonlinearity and electromechanical coupling. The NEMC (Nonlinear Electromagnetic Coupling) amplifies vibrations, notably affecting harmonic components with increased vibration, which is particularly prominent under the conditions of winding looseness. The research proposes using NEMCC (Nonlinear Electromagnetic Coupling Control) for condition detection, showing winding looseness variations, to limit harmonic EMF impacts. To address over-track and under-track problems in the SDF (Spectral Kurtosis-based Demodulation) process, Li et al. [88] presented the OSE (Optimal Symbolization Entropy) method. Because it can capture more fault-related information, the MSK index is recommended for partitioning method selection. Also, it suggests a multiscale OSE approach that combines Shannon entropy, optimal SDF, and coarse-graining techniques to identify bearing conditions. A test bench with a three-phase induction motor mechanically connected to a gearbox with different levels of gear wear—healthy, 25%, 50%, and 75% induced wear—was used by Elvira-Ortiz et al. [25] to validate its proposed method. The gear teeth diameters are uniformly altered to achieve different wear severities. One mechanical load that a variable frequency driver manages is a DC generator (VFD). Vibration data are obtained using a data collection system (DAS) with a 3 kHz sampling frequency and a triaxial accelerometer installed on the gearbox. Every test has four operating frequencies (5 Hz, 15 Hz, 50 Hz, and 60 Hz), lasts for 100 s, and collects 100 samples per wear condition for analysis. A water tank, a three-phase induction motor controlled by an inverter, and other components that change conditions such as cavitation and mechanical looseness comprise the test setup for analysing a flexible impeller pump in the work by Demircan et al. [103]. A three-axis accelerometer and an electrical current sensor are used for recording vibration and current signals, respectively. These signals are captured in a variety of conditions, including healthy and worn impellers, cavitation caused by throttling the inlet valve, and mechanical looseness caused by loosening a screw. The accelerometer, which is part of an EVAL-ADXL355 board, collects vibration data using SPI protocol before transmitting it to a computer over USB and collecting and timestamping the results with a Python script. The MCU, which was built on the STM32F4 discovery board, has been placed in charge of data acquisition. The accelerometer’s parameters include a 1 kHz low-pass filter cutoff frequency, a −3 dB point at 2.5 Hz for the integrated high-pass filter, and a measuring range of 8.192 g at a 4 kHz output data rate. At motor speeds ranging from 250 to 800 rpm, vibration signals have been collected in radial horizontal, radial vertical, and axial planes (x, y, and z axes). A clamp-type current sensor, which uses a transimpedance operational amplifier circuit architecture, tracks motor currents up to 30 A, while converting current to voltage and protecting ADC inputs from overvoltage with fast-switching schottky diodes. In RIT’s Compression Test Cell, a Dresser Rand piston compressor acted as the test platform in the work of Chesnes et al. [37]. This compressor possesses a single piston forcing cylinder on both sides and runs at 360 RPM. It is powered by a 10 hp motor. The primary focus is on using pressure transducers, an angular encoder on the crankshaft, and a triaxial accelerometer to monitor valve-spring parts. Using a LabVIEW interface, the CompactDAQ system gathers and analyzes data at 25.6 kHz. It has sensors such as an accelerometer (PCB 356A16) and pressure transducers (Omega PX309-100AI).

#### 6.1.6. Machine

Taking an experimental lift door system as a test case, Koutsoupakis et al. [5] presented an AI-based condition monitoring approach for identifying and detecting damage in mechanical systems. It shows how well an optimized Multibody Dynamics (MBD) model can simulate the system’s behavior and how parameter updating can be used to identify the system. Without additional optimization, the MBD models for faulty states, which are based on the optimal model, accurately simulate damage. Mystkowski et al. [8] monitored rotational speed by an E2B series inductive sensor, whose 24 DC voltage output was decreased for the microcontroller via a resistive divider. A four-channel data-processing system supporting ADC functions and statistical parameter calculations was built around an ATmega128 microcontroller. The resolution was improved to a maximum frequency of 62.5 kHz with a 2-bit ADC increase using methods such as interpolation, oversampling, and decimation. The system used IEPE sensors and a modified FFT technique for efficient operation, breaking up operations into discrete subroutines. A hydraulic FSW machine controlled by a PLC system was used in the experimental setup by Balachandar et al. [13] for monitoring tool conditions during friction stir welding (FSW). Aluminium 5083 plates were joined with non-consumable H13 tool steel, and vibration signals were recorded using a piezoelectric accelerometer attached to a data acquisition (DAQ) device (NI9234). The study evaluated vibration signals under different conditions, including a new tool, a terrible condition in which aluminium stuck to the tool, and a broken condition caused by improper handling or wrong RPM settings. The vibration signals were collected using a LabVIEW graphical program and a wireless four-channel DAQ device (NI 9234) at a sampling frequency of 51.2 kHz. Morgan et al. [113] conducted their experiments with the injection-molding machine CLF-60TX. A strain gauge and a PCB piezotronics accelerometer model 352A24 were used to collect the data. The IMC CRONOSflex system was used for collecting data. The cycle’s phases—closing, clamping, injection, and cooling—were fully recorded. Zonzini et al. [137] used an ACE Micromatic-Classic 20T CNC turning machine, a piezoelectric accelerometer attached to the tool holder, and a DACTRAN signal conditioning device in their experiment. A single-point carbide cutting tool was used on a mild steel shaft held in place by a pneumatic chuck. The accelerometer recorded vibration signals, which were converted to digital form by the DACTRAN device and stored on a computer via USB. The goal of this study was to monitor tool condition using vibration signals collected by the accelerometer.

### 6.2. Simulation

This section focuses on simulation, including research that uses advanced models to simulate real-world scenarios to monitor heavy machinery conditions.

#### 6.2.1. Gearbox

A Drivetrain Prognostics Simulator (DPS) was used by Koutsoupakis et al. [2] to examine the health of gear systems. An electric drive motor, planetary gear system, and two-stage gear system are all part of the configuration. The focus is on these gear systems, which use accelerometers to collect vibration data. The goal is to identify three states: one healthy and two damaged. The measurements are carried out at various RPMs with a constant load.

#### 6.2.2. Turbine

A 5 MW wind turbine’s detailed components, including the nacelle, gearbox, and several shafts, have been included by Amin et al. [16] in the study of the SIMPACK model. High-fidelity gear components are used in the gearbox to model gear tooth contact accurately. Nine wind situations with different wind loads and torques given to the nacelle were simulated without any gearbox issues. To simulate defects, two tooth pitch errors have been added to the model: one on the planetary gear and one on the high-speed shaft gear. Periodic impulses in vibration signals are caused by these flaws and are connected to the damaged gear’s rotating frequency. Different fault levels on individual teeth of the corresponding gears are represented by varying fault magnitudes (100, 50, and 20 microns). To identify and categorize these problems, the study analyzes vibration data from eight sensors installed on the gearbox. A 5 MW wind turbine is represented by the SIMPACK multibody model by Amin et al. [41], including the gearbox, drivetrain, and nacelle data. The drivetrain’s eight accelerometers record accelerations at various locations to resemble the behavior of a gearbox. Different wind conditions, such as turbulence and robust gust scenarios, are modeled to simulate real-world loads without gearbox failures. Simulated tooth pitch defects of various severity are created to simulate gear defects. The low-speed planetary stage and the high-speed shaft gear have these defects. Sensors for the low-speed stage and sensors for the high-speed shaft use vibration signals to detect and classify these defects.

#### 6.2.3. Bearing

Li et al. [88] created simulated fault signals for outer race, inner race, and ball faults by using the model equation for the N205 cylindrical roller bearing. When combined with white Gaussian noise, these artificial defects accurately represent situations with a 5 dB signal-to-noise ratio. They simulated the outer race, inner race, and ball faults as three different kinds of simulated bearing failure signals. Two simulated situations were used by Peeters et al. [130] to validate the proposed approach. The examples use cyclostationary source signals, one with periodic impulses and Gaussian noise and another with randomly spaced impulses to simulate bearing loss.

#### 6.2.4. Transport and Infrastructure

Tsunashima et al. [12] used SIMPACK a multibody dynamics software tool, to simulate the impact of railway track degradation on car-body vibrations. The simulation had been restricted to one ordinary railway vehicle. The model had seven rigid bodies, each with six degrees of freedom: one car body, two bogies, and four wheelsets. Appropriate joints connected the rigid bodies to simulate actual vehicle motion. The connection was made possible via spring and damper devices, each with spring constants and damping coefficients customised across three axial directions. It must be noted that this simulation study was carried out on a straight track. Fernando et al. [99] used both experimental and numerical analyses to determine the modal parameters, such as natural frequencies, mode shapes, and damping ratios, of two single-spanned pre-stressed concrete bridges: Bridge No 43/10 and Bridge No 13/3. Finite element models (FEMs) were generated by Patil et al. [131] with SAP2000 software used for numerical modal parameter identification. The FEM’s eigenvalue analysis showed dynamic features. To collect and analyze the structural responses generated by passing cars, the placements of the accelerometers, the measurement setup, and the specifics of the field vibration measurements were built correctly. This allowed for an in-depth examination of the bridges’ dynamic behavior over time. ANSYS was used by Nie et al. [132] to model a beam that is merely supported under stochastic excitations. Damage scenarios in which subelements near sensors are removed are given. Nine sensors measure acceleration and create information for healthy and damaged states.

### 6.3. Datasets

This section looks into studies that use existing datasets to find trends and patterns in monitoring heavy machinery conditions using vibration data.

#### 6.3.1. Gearbox

Balshaw et al. [1] used two different experimental datasets to evaluate their proposed model to show how responsive the latent manifold is to anomaly detection. A popular Condition Monitoring (CM) approach dataset is the IMS dataset from the NSF I/UCR Center for Intelligent Maintenance Systems. The vibration signals from three endurance tests, during which bearing defects developed, have been recorded in this dataset. Throughout the experiments, the experimental setup maintained the shaft speed at 2000 rpm and the weight at 6000 lbs constant. The Centre for Asset Integrity Management at the University of Pretoria has provided the experimental gearbox dataset, which is used as a standard to assess the effectiveness of the proposed Health Indicators (HIs) and Latent Health Indicators (LHIs). This dataset has several operating conditions using data for a gearbox in both healthy and unhealthy states. Two separate experiments were used to collect the data. First, 100 vibration samples were taken from the gearbox, which was considered to be in a healthy condition. After the gearbox had been disassembled, a specific gear tooth fault was introduced, which led to collecting 200 more vibration samples from the faulty condition. This central gearbox’s vibration samples were recorded for 20 s with a sampling rate of 25.6 kHz. The dataset, collected from a wind farm in northern Sweden from eighteen 2.5 MW Nordex N100 wind turbines, was used by Civera et al. [91] to validate the entropy-based Condition Monitoring method. An accelerometer placed on the output shaft bearing housing was used to collect vibration time-series data. A three-stage gearbox with sequential planetary gear stages and a helical gear stage was used in the turbine. Wang et al. [134] performed their experiment on three datasets. The first dataset used for analysis is bearing run-to-failure vibration data obtained from the University of Cincinnati’s Centre for Intelligent Maintenance Systems. This dataset comprises 984 vibration signal records collected at a sampling frequency of 20 kHz with each file containing 20,480 sampling points. The analysis focused on just healthy and faulty conditions for the purposes of bearing health monitoring and degradation evaluation in this study. They also used a gearbox run-to-failure dataset, which comprised 147 signal files, each containing 200,000 sampled points at a sampling frequency of 20 kHz. By increasing the load, the gearbox’s degradation process was accelerated. Using the Case Western Reserve University bearing dataset, they also evaluated their proposed model on bearing fault diagnosis. Two different datasets were used by Vos et al. [133]: the DST dataset (dataset-1) and the Airbus dataset (dataset-2). A two-stage helicopter gearbox endurance test created the DST dataset. During 30 s of the entire load operation, accelerometer measurements are collected from the ring gear of the gearbox at a 51.2 kHz sampling frequency. The Airbus dataset includes complex and variable vibration measurements from real-world helicopter flight tests. The dataset contains 1-min sequences of helicopter vibration levels recorded by accelerometers at 1024 Hz during flight tests. Half of the validation patterns are labeled abnormal, while the other half are labeled healthy. The absolute values in the dataset have been scaled for confidentiality reasons, making them meaningless in this scenario.

#### 6.3.2. Bearing

Aburakhia et al. [11] used the PU Bearing Dataset, CWRU Bearing Dataset and Ottawa Bearing Dataset for their research. Accelerometers were used on a 2 hp motor to record vibration data for the CWRU dataset. Different bearing components had different issues, and data at various motor speeds and loads were recorded. The dataset consists of signals from nine faulty operating conditions and healthy conditions to evaluate the method’s efficiency based on different system parameters and feature vector sizes. A 425-W PMSM running at a set speed and load recorded vibrations at a high sampling rate for the PU dataset. The entire set focuses on accelerated lifetime flaws and includes experimentally created faulty situations and healthy conditions. There are four classes with different kinds of faults in it. The University of Ottawa dataset recorded vibrations at a high sampling rate using a machinery malfunction simulator. The dataset includes different bearing health conditions and speed shifts, leading to 20 alternate configurations. The dataset is divided into one healthy class and four faulty classes based on bearing conditions and speed changes. Ahmad et al. [139] used two datasets: one from rotating machine data and the other from bearing data. The bearing dataset consists of vibration signals from four bearings divided into three sets. This dataset contains the data of independent test-to-failure experiments. The dataset from rotating machines contains data collected from five different rotating machines, each of which is of the same type but is manufactured differently. They consist of three-dimensional vibration signals recorded by accelerometers attached to each RM’s housing. Peeters et al. [130] proposed a model they built using vibration signals from NASA’s Prognostics Data Repository obtained from the University of Cincinnati’s Center for Intelligent Maintenance Systems (IMS). This dataset collected the run-to-failure condition of an outer race fault in a bearing. Four Rexnord ZA-2115 bearings, as well as high-quality accelerometers, were installed on the shaft. Zhao et al. [138] used the CWRU dataset, including generating defects on the motor’s bearings at different positions. The dimensions of these flaws, which are called outer raceway (OR), rolling element (RE), and inner raceway (IR), varied from 0.1778 to 0.7112 mm. Twelve different bearing fault types were the primary goal of this diagnosis. The obtained one-dimensional data were segmented using a segment length of 256 and a sliding step of 64 to find a balance between computational cost and data capacity. For each class, approximately 1800 segments were generated. These segments went through the continuous wavelet transform (CWT), which produced a 256 by 256 matrix of wavelet coefficients, which was then converted to grayscale images. Brusa et al. [136] used the vibration dataset and collected this from the test rig. The test rig for this study was created for industrial medium-sized bearings at Politecnico di Torino’s Mechanical Engineering Laboratory. The experimental setup drives the shaft up to 2500 rpm using a 30 kW three-phase SIEMENS motor attached to a SIEMENS G120_CU240E_2 INVERTER with a brake resistor. SKF CMSS 2200 T sensors provides vibration data, which the LMS Scadas III Digital Acquisition system collects. SKF spherical self-aligning bearings were installed on the rig for the vibration dataset. Vibration signals recorded at a sampling frequency of 20,480 Hz were initially processed to reduce noise. Three defect conditions—inner race (IR) and outer race (OR) defects—were introduced separately, and a range of radial loads and rotational speeds are covered in the dataset. Yin et al. [135] made use of the Case Western Reserve University (CWRU) bearing fault dataset, which focuses on inner and outer raceway faults (ORF) in induction motors with bearing defects. Each fault mode has three defect sizes: 0.1178, 0.3556, and 0.5334 mm. Data were acquired using an accelerometer placed on top of the motor’s case and sampled at 48 kHz for approximately 10 s. The BPFO (ball pass frequency outer) and BPFI (ball pass frequency inner) fault frequency characteristics are calculated mathematically. These frequencies are calculated using various factors such as rotation frequency, bearing geometry, and rolling element contact angle. Li et al. [88] use the experimental 6203 bearings dataset from Paderborn University to evaluate how well the proposed multiscale OSE (Optimal Scale Entropy) classifies different rolling-bearing health conditions. A 425 W permanent magnet synchronous motor running at a particular torque, speed, and current specification is part of the experimental setup. The values given correspond to the following parameters: radial force, torque applied to the load, sampling frequency, and rotating speed: 1500 rpm, 400 N, 0.7 N m, and 64,000 Hz. The dataset includes five different types of artificially induced bearing faults for validation through accelerated lifetime tests. There are 25 testing samples and 75 training samples for each kind of fault. The IMS dataset is divided into three distinct datasets in work by Afridi et al. [21], each representing a separate run-to-failure experiment. Dataset 02, which includes 984 files, was chosen for model training and testing. This dataset was divided into training and testing sets, with 70% given to training the model and 30% kept for testing its performance. Furthermore, the model’s performance was validated using Dataset 03, which contained 4448 files. In particular, at the end of their respective run-to-failure experiments, Datasets 02 and 03 recorded outer race faults in bearings. After that, the model trained and tested on Dataset 02 was then verified against Dataset 03 to determine its reliability and generalizability in detecting these defects.

#### 6.3.3. Transport and Infrastructure

The publicly available Z24 bridge dataset was used by Zonzini et al. [137]. Eleven accelerometers and environmental sensors monitored the Z24 bridge for a whole year, gathering real-time operational data as well as intentionally creating breakdown events. The accelerometers made 11-min observational windows by gathering 65,536 acceleration measurements at 100 Hz every hour. It analyzes the effects of improved compressed sensing on the detection of bridge health, uses temperature data as a direct input for neural network models, and accounts for instrumental flaws in the monitoring procedure, particularly the noise density in MEMS accelerometers.

## 7. Conclusions and Recommendation for Future Work

### 7.1. Conclusions

We carried out a comprehensive review of vibration-based condition monitoring. We searched the ‘vibration-based condition monitoring’ keyword on Google Scholar for relevant academic papers. We collected a list of the top 40 papers on vibration-based condition monitoring published between 2020 and 2023 for an in-depth overview of the most recent advances. For the quality and relevance of the selection, we excluded papers that were irrelevant to vibration-based condition monitoring. The papers reviewed cover various vibration data-gathering sources, including gearboxes, bearings, turbines, and machines, and use approaches such as simulations and pre-existing datasets. Most papers used accelerometers as the primary data collection method, indicating that they provide a primary sensor in vibration collection. The machinery and its vibration characteristics determine the choice of accelerometer type along with systematic requirements. Based on these requirements, researchers select wireless/wired sensors, accelerometer technologies and measurement axes, uniaxial or triaxial measurement, and different technologies such as piezoelectric, highlighting the importance of matching the sensor’s capabilities to the specific demands of the vibration analysis for monitoring. A few papers used simulations or existing datasets to collect vibration data, showing different vibration-based condition monitoring research techniques. Simulating real-world environments with software is considered appropriate if it accurately replicates the required situations, making it an ideal strategy for vibration-based condition monitoring research in some scenarios.

### 7.2. Recommendation for Future

#### 7.2.1. Miniaturization

Integrating smaller accelerometers into machinery is easier, particularly in small locations where larger sensors cannot fit. This improvement will enable more applications and more flexible placement.

#### 7.2.2. Wireless Technology

Real-time monitoring has become possible without being restricted by wired connections due to improved wireless capabilities. This is important for effectively relocating sensors across heavy equipment components.

#### 7.2.3. Power Efficiency

Better power efficiency is significant for battery-powered accelerometers. Improving battery life or allowing renewable energy sources minimizes maintenance requirements while ensuring ongoing monitoring.

#### 7.2.4. High-Frequency Range

High-frequency vibrations generally indicate heavy machinery defects. Increasing the frequency range of the accelerometers means that possible defects, particularly during the early phases, are detected and addressed quickly.

#### 7.2.5. Durability

The surroundings of heavy machinery are dangerous. Accelerometers can be made more robust and durable by facing shocks, vibrations, and environmental conditions, extending their lifespan and accuracy.

#### 7.2.6. Advanced Materials

To ensure that even tiny vibrations are precisely detected and recorded, advanced components can be used in sensor manufacturing to increase sensitivity and accuracy.

#### 7.2.7. Temperature Stability

Heavy machinery works in various conditions and temperatures. Accelerometers with higher temperature stability enable consistent and accurate performance in various operational environments.

## Figures and Tables

**Table 1:** Accuracy of recorded data by accelerometers.

Accelerometer	Accuracy
Piezoelectric accelerometer	High level of accuracy for high-frequency vibrations. The lower frequencies have a limited accuracy.
Piezoelectric uniaxial accelerometer	Maintain accurate measurements along one axis to ensure accuracy in that direction.
PCB piezoelectric accelerometer	Overall provides good accuracy for various vibration measurements.
Wired and wireless accelerometer	Although the mode of transmission has no direct impact on accuracy, wireless systems may experience interference problems.
Acoustic emission transducers	Accuracy may differ from accelerometers.
MEMS accelerometer	When compared to larger, more specialised accelerometers, they may have slightly lower accuracy.
MEMS dual-channel accelerometer	Accurately detects vibrations along two axes, with multi-axis capability.
Stud-mounted single-axial accelerometer	Accuracy depends on accurate installation.
Magnetic triaxial accelerators	Effective over a broad frequency range but may be subjected to external magnetic fields.
Triaxial accelerometer	Relatively lower in accuracy than single-axis accelerometers.

**Table 2:** Review of research paper simulation software.

Refs.	Simulation Software	Description
Koutsoupakis et al. [2]	Drivetrain Prognostic Simulator by Spectra Quest Inc.	Multibody dynamics system
Naveen Venkatesh et al. [3]	Numerical Simulation	A gearbox multivariate vibration signal was simulated as a six-channel multivariate synthetic signal as a set of equations
Tsunashima et al. [12]	SIMPACK	To detect and isolate the track faults from car-body vibration
Amin et al. [41]	SIMPACK	Simulated an onshore 5 MW three-blade up-wind turbine model
Li et al. [88]	Not Mentioned	They simulated the fault signals by their equations
Fernando et al. [99]	Numerical Simulation	Establish the level of existence of damages in the studied PC bridges
Amin et al. [16]	SIMPACK	Full wind turbine simulations with varying wind conditions using a SIMPACK multibody model of a 5 MW (MW) wind turbine
Kamariotis et al. [129]	Simulator	The model is used as a simulator for creating dynamic response measurement samples from the bridge system
Peeters et al. [130]	Not Mentioned	Periodic impulses with Gaussian distributed amplitudes and randomly spaced impulses representing the slip in bearings
Patil et al. [131]	Numerical Simulation	Simulating the dynamic condition of bearings
Nie et al. [132]	ANSYS	Simply supported beam under stochastic excitations is simulated

**Table 3:** Refs., Datasets, and Descriptions.

Paper	Dataset	Dataset Collected from
Balshaw et al. [1]	IMS dataset	Bearings
Aburakhia et al. [11]	Case Western Reserve University (CWRU) bearing dataset	Bearing
Afridi et al. [21]	Prognostic Data Repository of NASA	Bearings
Li et al. [88]	DST dataset Airbus dataset	Helicopter main gearbox Helicopter flight test
Civera et al. [91]	Dataset collected from wind turbine	Northern Sweden and consisting of 18 2.5 MW Nordex N100 wind turbines
Peeters et al. [130]	IMS dataset	Bearings
Vos et al. [133]	DST dataset Airbus dataset	Helicopter main gearbox Helicopter flight test vibration measurements
Wang et al. [134]	Vibration dataset Gearbox run-to-failure dataset Case Western Reserve University dataset	Accelerated bearing degradation analysis Accelerated gearbox degradation analysis Bearing
Yin et al. [135]	CWRU Dataset	Case Western Reserve University gathered on an induction motor with faults in the bearings.
	SCPB Dataset	SCP bearing (SCPB) dataset was gathered for an industrial submersible centrifugal pump with bearing faults.
Brusa et al. [136]	Mechanical Engineering Laboratory of Politecnico di Torino	Medium-sized bearings
Zonzini et al. [137]	Z24-Bridge Dataset	Z24 bridge
Zhao et al. [138]	Dataset from Case Western Reserve University XJTU-SY	Bearings Bearings
Ahmad et al. [139]	IMS bearing dataset Private industry dataset	Bearing Rotating machinery

**Table 4:** Data collection machines used in the literature.

Refs.	Machine
Koutsoupakis et al. [5]	Elevator door rail
Mystkowski et al. [8]	Soft winding machine Carding machine
Balachandar et al. [13]	Reciprocating compressors
Mehamud et al. [14]	Conventional lathe machine
Mongia et al. [27]	Centrifugal pumps
Feng et al. [35]	Injection molding machine
Patil et al. [39]	Stir welding machine
Civera et al. [91]	ZDJ9 point machine
Mukherjee et al. [98]	Industrial VMC
Fernando et al. [99]	Milling machines
Lin et al. [100]	CNC Machine center
Kamariotis et al. [129]	Spectra Quest Machinery
Espinoza-Sepulveda et al. [145]	Automobile brakes
Yaghoubi et al. [148]	FSW machine
Espinoza Sepúlveda et al. [151]	CLF 600TX Plastic Injection Molding Machine
Shi et al. [156]	Sieving screen
Morgan et al. [157]	RUB-Tunneling Device

## Data Availability

Not applicable.

## Abbreviations

The following abbreviations are used in this manuscript:

CNC	Computer Numerical Control
CTC	Capacitive-to-Digital Converter
DAQ	Data Acquisition Device
DAS	Data Acquisition System
IM	Inducton Motor
MCU	Microcontroller Unit
MEMS	Micro-Electro-Mechanical Systems
PCB	Printed Circuit Board
PGB	Parallel Gearbox
SHM	Structural Health Monitoring
PLC	Programmable Logic Controller
